# Mid‐manufacturing storage: Antibody stability after chromatography and precipitation based capture steps

**DOI:** 10.1002/btpr.2928

**Published:** 2019-11-01

**Authors:** Walpurga Krepper, Daniel Burgstaller, Alois Jungbauer, Peter Satzer

**Affiliations:** ^1^ Department of Biotechnology University of Natural Resources and Life Sciences Vienna Austria; ^2^ Austrian Centre of Industrial Biotechnology (ACIB) Vienna Austria

**Keywords:** chromatography, immunoglobulin, precipitation, storability, storage

## Abstract

Antibodies of the IgG2 subclass were captured from the clarified cell culture fluid either by protein A chromatography or by polyethylene glycol precipitation. The captured intermediates were stored as neutralized eluates (protein A chromatography) or in solid form as polyethylene glycol precipitates over a period of 13 months at three temperatures, −20°C, 5°C, and room temperature to compare the capture technologies in regard of the resulting product storability. Monomer content, high molecular mass impurities product loss and changes in the composition of the charge variants were determined at six time points during the storage. At the beginning and end of the study, samples were additionally tested by differential scanning calorimetry, differential scanning fluorimetry, and circular dichroism to determine structural alterations occurring during storage. Protein A purified material was highly stable at all tested temperatures in regard of monomer content and product losses. A transient, acidic isoform was formed during the chromatography step which re‐converted to the main charged variant upon storage within a matter of days. Precipitated antibodies could be stored at −20 or 5°C for 3 months without product losses but afterwards recovery yields dropped to 65%. At room temperature, the precipitated antibody was not stable and degraded within 3 months.

## 1 INTRODUCTION

Stability and shelf life of formulated antibodies have been studied extensively but there is limited data available for process intermediates. Research is classically based on ideal storage conditions for final drug formulations^1^, ^2^, ^3^ but nowadays biopharmaceutical operations are often conducted on a global scale and the materials might be shipped mid‐manufacturing. In 2016, the European Medicine Agency (EMA) published a guideline on process validation for the manufacturing of biotechnology‐derived products^4^ where they recommend to evaluate the impact of hold steps, mid‐manufacturing storage, and transportation on process *intermediates*. To determine the influence of unexpected process interruptions on the intermediate, they suggest to perform studies under worst‐case and non‐standard conditions. Furthermore, intermediate characterization is crucial for process development, design of hold steps, and development of in‐process control methods. Therefore, protein alterations and stability at all stages during processing are important.

Stability studies are time and cost consuming, since the samples must be stored under the respective temperature and conditions for the projected time period to get good quality data. Accelerated shelf‐life studies have been suggested in which the sample is stored at higher temperature and then possible storage time at lower temperature is extrapolated using the Arrhenius equation.^5^ For elaboration of shelf‐life some quality parameters reflecting the degradation of the protein must be determined.

All proteins, including recombinant antibodies, are susceptible to alterations during production, processing, and storage. Common modifications of antibodies are sialylation, glycosylation, deamidation, oxidation, N‐terminal pyroglutamine cyclization, C‐terminal lysine cleavage, aspartate isomerization, and disulfide bond shuffling.^6^ These modifications give rise to a high degree of microheterogeneity and can affect antibody functionality and stability.^7^ It is not possible to capture all these variants therefore surrogate parameters are used during product and process development. Importance has however to be put on the identification of critical protein modifications in order to limit degradation pathways and define optimum storage conditions in regard of temperature, humidity, and light irradiation.

Rapid methods to investigate antibody destruction during storage are usually HPLC based with the most widely used techniques being size exclusion chromatography, cation‐exchange chromatography or content analysis by affinity chromatography. Such methods capture the aggregate formation, degradation/clipping of the antibody to a smaller molecular variant and formation of charge variants.

Nowadays, the majority of antibody products is produced in mammalian host cell lines and after primary recovery, the purification is based on protein A chromatography which typically results in yields around 95%^8^ and purities >98%.^9^ Due to the high affinity of protein A towards antibodies and the high capacities achieved by modern protein A materials, the protein solution is rather concentrated and pure at this stage. The high antibody concentration in combination with the harsh, acidic elution conditions can promote the formation of aggregates and therefore potentially to a loss in product.^10^ Also, protein A chromatography material is rather expensive, adding significantly to the overall process costs, especially in early development phases when the material is not utilized to its maximum lifetime.^11^ Therefore, several groups – including our own – have presented alternative approaches for antibody capture steps in recent years, for example, by mixed‐mode chromatography,^12^ aqueous two‐phase extraction^13^ or precipitation.^14^, ^15^, ^16^ Precipitation offers a viable, non‐chromatographic alternative which can be easily customized for continuous manufacturing. It scales only with volume and is able to deal with varying titers in the upstream feed. Burgstaller et al. recently demonstrated that polyethylene glycol (PEG) precipitation in combination with tangential microfiltration results in similar purity and process yields as protein A chromatography.^17^ In a PEG precipitation capture step, the solubility of the antibody in the clarified culture supernatant is lowered by addition of PEG leading to precipitation of the product. The solid precipitate is harvested by microfiltration and resolubilized in a dissolution buffer. The antibody is thereby concentrated and host cell proteins (HCPs) are largely depleted. To meet the requirements in regard of purity for drug products, the protein has to be further processed by at least one more polishing step before it will be transferred into a formulation buffer by an ultra‐/diafiltration step.

In this work, we compared two antibody capture technologies in regard of effects on the intermediate storability. Antibodies were captured either by protein A chromatography or by precipitation from the clarified cell culture fluid. The protein A eluate was neutralized and stored in liquid phase. The precipitated material was stored in its solid form after decantation of the supernatant. Samples were then stored under identical conditions at three temperatures (−20°C, 5°C, and room temperature) for 13 months. Storage at −20 and 5°C are typical storage conditions for biopharmaceutical intermediates awaiting further processing. Storage at room temperature (ambient, non‐monitored) was included as a worst‐case scenario. The EMA guideline for process validation^4^ recommends tests at higher temperatures and for elongated times to substantiate the recommendations for ideal intermediate handling during manufacturing. In both cases, the material was stored mid‐manufacturing without performing any additional processing steps before storage. It should be noted that this results in different high molecular weight impurities (HMWI) and low molecular weight impurities (LMWI) concentrations for the two pools since protein A chromatography is more efficient in HCP clearance. The precipitated material is stored under precipitation conditions, that is, 13.2% (w/w) of polyethylene glycol are present. Storage of the material in its precipitated form poses the advantages that less volume is being stored which leads to a decrease in storage costs.

The reconstituted material was tested by HPLC based methods (size exclusion and cation exchange pH gradient) at six time points during storage (starting material and after 1, 3, 6, 10, and 13 months at the respective temperature). Starting and end material were additionally probed by differential scanning calorimetry (DSC), differential scanning fluorimetry (DSF), and circular dichroism (CD) to identify structural changes during storage.

## 2 MATERIAL AND METHODS

All chemicals were of analytical grade and purchased from Sigma‐Aldrich (St. Louis, MO), unless stated otherwise.

### 2.1 Antibodies

Experiments were carried out with recombinant, human antibodies of the subclass IgG2. We used clarified cell culture fluid (CCCF) of a CHO run with an antibody monomer concentration of 3.3 g/L. The antibodies were then either captured by protein A chromatography (chapter 2.1.1) or precipitation (chapter 2.1.2) using CCCF from the same CHO run.

#### 2.1.1 Protein A chromatography

Protein A chromatography was carried out on Mab Select SuRe resin (GE Healthcare, Sweden). Column was equilibrated with 50 mM Na‐phosphate at pH 7.0 and washing was performed with 100 mM Na‐citrate at pH 5.5. Antibodies were eluted by a single step gradient using 100 mM Na‐acetate at pH 3.6. They were then incubated at pH 3.6 for 1 hr before the pH was readjusted to 6.0.

#### 2.1.2 Precipitation

Polyethylene glycol with an average molecular weight of 6,000 Da was used for antibody precipitation as described in previous publications.^14^ In short, a stock solution of 40% PEG6000 (w/w) was prepared in a 100 mM Tris buffer (pH 7.5). The CCCF was mixed in a polypropylene tube with the stock solution to a final PEG concentration of 13.2% (w/w). Then, the solution was mixed on a mechanical end‐over‐end shaker (Cole‐Parmer, IL) for 10 min. The precipitate was centrifuged for 5 min at 2,000 rcf and the liquid phase decanted. The precipitate was washed with a solution of 13.2% PEG in 100 mM Tris (pH 7.5) (gravimetrically determined to be the same amount as previously removed) and again incubated on the shaker for 10 min. After another centrifugation (5 min at 2,000 rcf), the CCCF was decanted and the precipitate was put into storage. Before analysis, the precipitated antibody was reconstituted in the same volume previously removed by decantation. As reconstitution buffer, 100 mM Tris–HCl buffer (pH 7.5) was used. The protocol and screening for precipitation conditions was already presented in more detail by Burgstaller et al.^17^

### 2.2 Storage conditions

In both cases, protein A purification and precipitation, the material was stored without performing any additional process steps. The protein A purified material was stored in liquid form after neutralization. The precipitated material was stored in the precipitated form after removal of the supernatant. Conical, lightproof polypropylene tubes were used for storage. Storage at −20°C was done in a standard lab freezer from Electrolux (Stockholm, Sweden) and storage at 5 ± 3°C was done in a refrigerator by KBS Kältetechnik (Wiesbaden, Germany). Room temperature storage was performed in one of our labs with room temperature ranging from 20.0 to 25.0°C. This scenario was included to mimic worst‐case conditions like an unexpected process interruption where fast degradation kinetics can be expected.

### 2.3 Nano differential scanning calorimetry (nanoDSC)

Samples were dialyzed in Slide‐A‐Lyzer cassettes (Thermo Fisher Scientific, Waltham, USA) with a molecular weight cut‐off (MWCO) of 10.000 Da to a 20 mM sodium phosphate buffer, pH 6.9 and then diluted to a concentration of 3 mg/ml. The solution was loaded into the sample cell of a TA‐Instruments (New Castle, DE) Nano DSC instrument (model: 602000). The reference cell was filled with a 20 mM sodium phosphate buffer pH 6.9 and a thermoscan from 20 to 100°C with a scan rate of 1°C/min was performed. In between sample runs, the instrument was cleaned by flushing the cells with water and buffer. At the end of each set of experiments, the instrument was cleaned with a solution containing 0.5 M NaCl, 0.1 M acetic acid, and 1 mg/mL pepsin which was incubated for 3 hr at 37°C and then flushed with 2 L of water. The obtained thermogram data were analyzed using the TA Instruments NanoAnalyse software. Buffer blanks were subtracted from all samples.

### 2.4 Circular dichroism (CD)

Circular dichroism spectra were obtained on a Chirascan CD Spectrometer from Applied Photophysics (Surrey, UK). The samples were diluted to concentrations of 0.3 g/L and measured in cells with a path length of 0.1 cm. Spectra were obtained in the range of 180 to 260 nm. The bandwidth was set to 1 nm and the signal was averaged over 10 s. The detector reached saturation at 195 nm, therefore, data in the range from 195 to 260 nm is shown.

### 2.5 Size exclusion chromatography (SEC)

SEC was used to assess the monomer and HMWI content and product losses. Experiments were carried out on a Dionex UltiMate 3000 HPLC system (Thermo Fisher Scientific, Waltham). Isocratic elution was carried out with a 50 mM sodium phosphate buffer with 150 mM NaCl at pH 7.0. 10 μl of 0.22 μm filtered sample were applied to a TSKgel® G3000SWXL HPLC column (5 μm; 7.8 mm × 300 mm) with a TSKgel® SWXL guard column (7 μm; 6.0 × 40 mm) (both Tosoh, Japan). Chromeleon™ 7 software (Thermo Scientific) was used to monitor the signals at 215 nm (for HMWI) and 280 nm (for monomer content and product loss). The starting material is referred to as *T*_0_ material. Monomer content is based on the ratio of product peak area (monomer, detected at 280 nm) to the sum of all peak areas. HMWI are calculated by taking the sum of all peaks eluting before the main antibody peak (at 215 nm) and dividing it by the sum of main peak and earlier eluting species. Concentration in relation to *T*_0_ is the ratio between the monomer peak at 280 nm of the respective sample with the *T*_0_ sample of the respective capture method (protein A or precipitation). Average values and standard deviations are based on triplicate measurements. Injection volume and dilutions were kept constant for all samples.

### 2.6 Isoform characterization by pH gradient chromatography

Antibody isoforms were monitored by a cation exchange (CIEX) HPLC method based on a pH gradient method developed by Lingg et al.^18^ In short, measurements were performed on a Dionex UltiMate 3000 HPLC system (Thermo Scientific). A ProPac™ WCX‐10G Guard Column (10 μm, 4 × 50 mm) and a ProPac™ WCX‐10 column (10 μm, 4 × 250 mm) (both Thermo Scientific) were used as stationary phase. Samples were diluted to 1 g/L and the injection volume was 100 μl. The flow rate of the method was set to 1 ml/min. The column was equilibrated for 1 CV at 100% A, before a 12 CV gradient from 0 to 100% B was performed. The method ended with a 1 CV wash step at 100% B. Mobile phase A was 5.5 mM HEPES, 4.2 mM Bicine, 9.5 mM CAPSO, 0.8 mM CAPS and 6.3 mM NaCl (pH 8.0). Mobile phase B was 10.5 mM Bicine, 2.5 mM CAPSO, 7.0 mM CAPS (pH 10.5). The outlet was monitored at 280 nm. The most abundant isoform is defined as main charge variant (MCV). Isoforms eluting earlier than the MCV are called acidic variants, later eluting ones are called basic variants. Accuracy, precision and the lower limit of quantification of the method can be found at Lingg et al.^19^ Injection volume and dilutions were kept constant for all samples.

### 2.7 Nano differential scanning fluorimetry (nanoDSF)

Nano differential scanning fluorimetry measurements were performed on a Tycho NT.6 (Nanotemper, Germany). Samples were diluted with 25 mM Tris (pH 7.5) to a concentration of 1 g/L and taken up in glass capillaries which were placed in the nano differential scanning fluorimeter. Initial temperature was set to 35°C and the ramp‐up rate was set to 30°C/min. Spectra were taken between 35 and 95°C.

## 3 RESULTS

### 3.1 Monomer content, HMWI, and product loss

**Figure 1:**
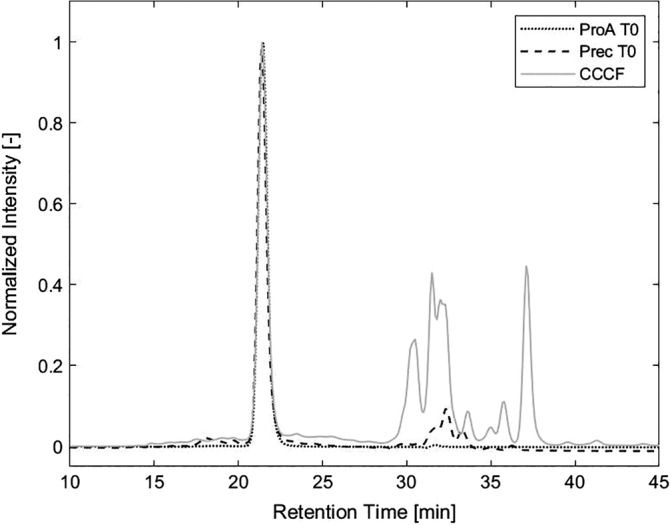
Size exclusion chromatogram of the antibodies before (CCCF) and after capture step (either protein A chromatography or precipitation). Due to different purification efficiencies, the amount of HMWI and LMWI are lower in the protein A purified pool compared to the precipitated material. CCCF, clarified cell culture fluid; HMWI, high molecular weight impurities; LMWI, low molecular weight impurities

To determine the stability of the antibody intermediate, we used clarified cell culture supernatant and purified it either by a conventional protein A chromatography step or by precipitation of the antibody with PEG at a concentration of 13.2% (w/w). The protein A eluate was kept at the elution pH of 3.6 for 1 hr and then readjusted to the storage pH of 6.0. The PEG precipitated sample was stored in its solid form and in the presence of 13.2% polyethylene glycol. To simulate different intermediate storage conditions, samples were stored at three different temperatures. Storage at −20 and 5°C corresponds to typical storage conditions mid‐manufacturing while storage at room temperature was included as a worst‐case condition where rapid degradation kinetics are expected. The material was stored for a total of 13 months. Multiple aliquots for both protein A purified and precipitated material were stored at these temperatures and for each analysis an independent aliquot was used and discarded after analysis to avoid repeated freeze–thaw cycles of samples. Samples were tested at the start and after 1, 3, 6, 10, and 13 months in storage with different methods for monomer content, aggregates and product losses. Changes in protein structure were determined by CD, DSC, and DSF. Figure 1 shows the composition of the samples before and after capture step. HMWI as well as LMWI present in the CCCF are more efficiently removed by protein A chromatography than by precipitation. For further information regarding the efficiency of precipitation methods in comparison to affinity chromatography, see Sommer et al.^14^, ^20^ and Hammerschmidt et al.^15^

**Figure 2:**
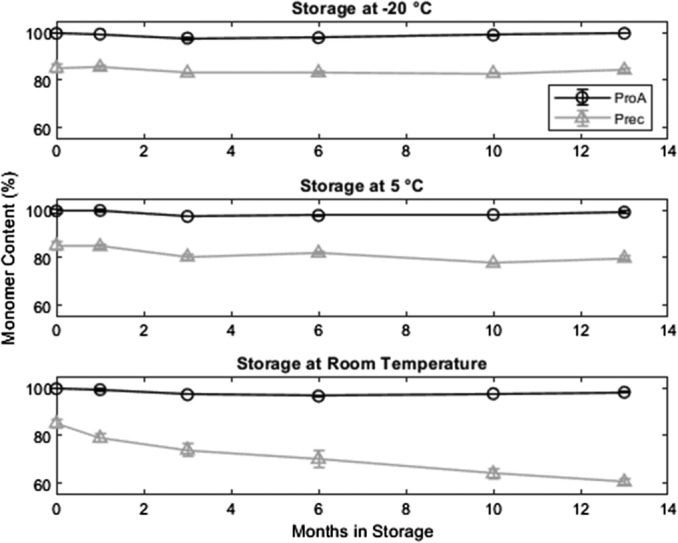
Monomer content of protein A purified and precipitated material at three storage temperatures. For protein A purified material, the monomer content is stable at all tested temperatures. For the precipitate, the monomer content is stable at −20 and 5°C but decreases when stored at room temperature. Error bars are based on triplicate measurements

**Figure 3:**
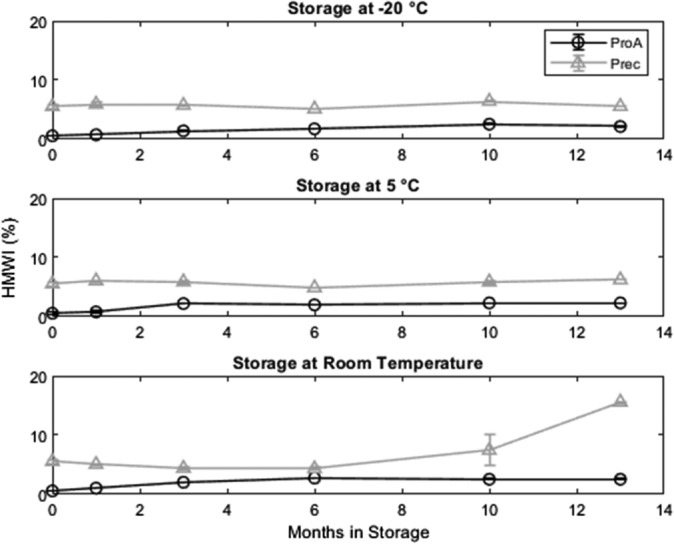
HMWI content in protein A purified and precipitated material at three storage temperatures. For protein A purified material, the HMWI stay constant at all tested temperatures. In the precipitated material, the HMWI concentration is significantly higher compared to the protein A purified material. When stored at −20 or 5°C, the HMWI content in the precipitated sample remains stable. At room temperature, the HMWI of the precipitated material accumulate over time. Error bars are based on triplicate measurements. HMWI, high molecular weight impurities

Figure 2 gives the monomer content for both purification techniques at the three tested temperatures. Over the testing period of 13 months, the monomer content of the protein A purified samples decreases approximately 2% from 99.8% at *T*_0_ to ~98% at all tested temperatures. This decrease is mirrored by a rise in HMWI (Figure 3).

For the precipitated antibody, the monomer content (purity) stays constant at −20°C, decreases slightly at 5°C and strongly when stored at room temperature. For the material stored at 5°C, the monomer loss is counteracted by an increase in HMWI. The monomer content of the precipitated material decreases at a constant rate over the observation period. However, HMWI stay constant for the first 6 months in storage and start rising only afterwards. Therefore, the monomer loss of the first 6 months has to be the result of degradation processes leading to the formation of LMWI. Generally, the HMWI content in the precipitated samples is around 5% at the start of the storage, while it is less than 1% for the protein A purified samples.

**Figure 4:**
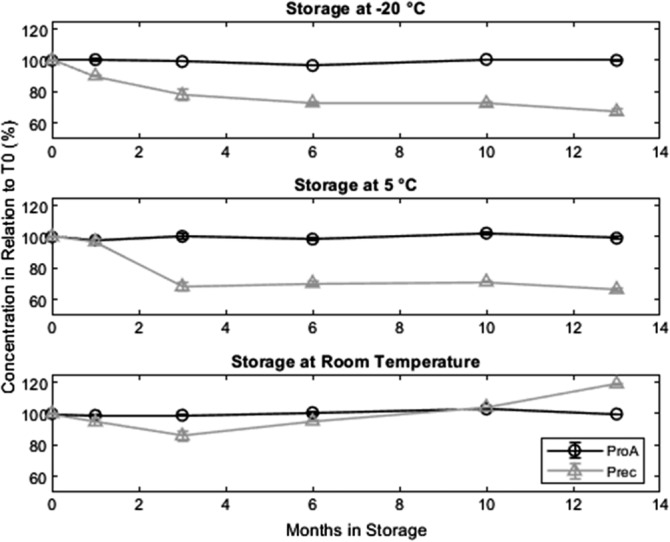
Concentration of protein A purified material and precipitated material in relation to the concentration at *T*_0_. Yields for the protein A purified material are stable at all tested temperatures. The precipitated material stored at −20 or 5°C needs to be reconstituted within the first 3 months of storage, otherwise yields drop to 65% where they stay constant. Precipitated material stored at RT is degraded as shown by CIEX‐HPLC (Figure 5b). Error bars are based on triplicate measurements

We do not observe any product losses for the protein A purified materials at any temperature (Figure 4). For the precipitated material, the picture looks quite different. The product loss of the material stored at −20 and 5°C is less than 10% if reconstituted within 1 month but if the precipitate is stored longer than that, the losses increase to 30 to 35%. To keep recovery yields high, it is therefore necessary to reconstitute the precipitated material within the first month of storage.

**Figure 5:**
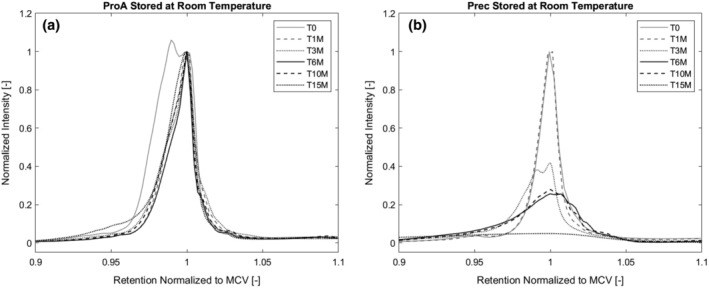
(a) Isoform pattern of protein A purified material and (b) precipitated material stored at room temperature. Acidic variant of protein A purified material reconverts to MCV within first month of storage. Precipitated material is not stable at room temperature, the formation of various new isoforms can be observed during storage. MCV, main charge variant

For the precipitated material stored at room temperature, we observe a small product loss during the first 3 months of storage, afterwards the SEC HPLC data indicate no product losses resulting in resolubilization yields above 100% [sic] after 13 months. While the overall yield is stable at 100%, the product is unstable at room temperature. We assume degradation into product related impurities (degradation products) as the yield is stable, but the monomer content of the sample drops down to 60% during storage at room temperature. Due to the results regarding monomer content (Figure 2), HMWI (Figure 3), and charged variants (Figure 5), we assume that the antibody is degraded after 13 months of room temperature storage and that the degraded antibody has a higher UV absorbance than the native form explaining a yield above 100%. Since obtaining product losses less than 0% is virtually impossible, we assume that tryptophan residues which are buried in the hydrophobic core of the native protein are becoming exposed to the hydrophilic solvent due to the loss of conformation and therefore increase the UV signal. The data shown in Figures 2 and 3 is presented in tabular form in as well (Table S1).

### 3.2 Acidic variants formed during protein a chromatography

#### 3.2.1 Variant alterations during storage

**Figure 6:**
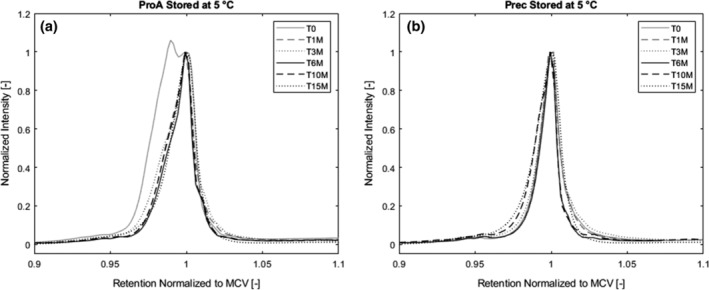
(a) Isoform pattern of protein A purified material and (b) precipitated material stored at 5°C. Acidic variant of protein A purified material reconverts to MCV within first month of storage. Precipitated material has a stable isoform pattern. MCV, main charge variant

Isoform composition of the stored material was determined at six time points during storage of 13 months for the materials stored at −20°C, 5°C, and room temperature to catch any changes in the isoform composition indicating possible degradation of the antibody during storage. The *T*_0_ sample of the protein A purified material was additionally tested for isoform pattern changes in the first 120 hr of storage at temperatures 5°C, room temperature, and 40°C since we detected an acidic variant in the starting material that vanished in storage within the first month. We hypothesized that the formation of the acidic variant could be a result of the low pH hold step after protein A elution, therefore we prepared a sample of precipitated material resolubilized and stored for 1 hr at low pH to capture any changes in isoform composition which are not due to the protein A purification itself, but due to the following low pH viral inactivation. Figure 6 shows a pH gradient HPLC chromatogram of the antibody before capture of clarified cell culture fluid (CCCF), the samples of the protein A purified material and the precipitated material after low pH incubation before storage.

**Figure 7:**
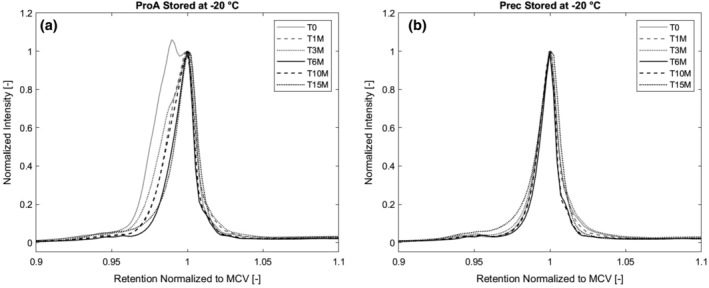
(a) Isoform pattern of protein A purified material and (b) precipitated material stored at −20°C. Acidic variant of protein A purified material reconverts to MCV within first month of storage. Precipitated material has a stable isoform pattern. MCV, main charge variant

Protein A chromatography leads to the formation of a new isoform in the sample which is more acidic than the one present in the CCCF while precipitation or viral inactivation at low pH does not result in formation of any new variants. In this chapter, we will however focus on the alterations during long‐term storage while the formation of the acidic variant during protein A chromatography is dealt with in more detail in chapter 3.2.2. Figure 7a shows the protein A purified material during long term storage at −20°C. As we can see, the acidic variant is converted into the MCV within the first month of storage. Afterwards, the isoform pattern is stable again. The precipitated antibody stored at −20°C depicts a stable isoform pattern over the monitoring period (Figure 7b).

**Figure 8:**
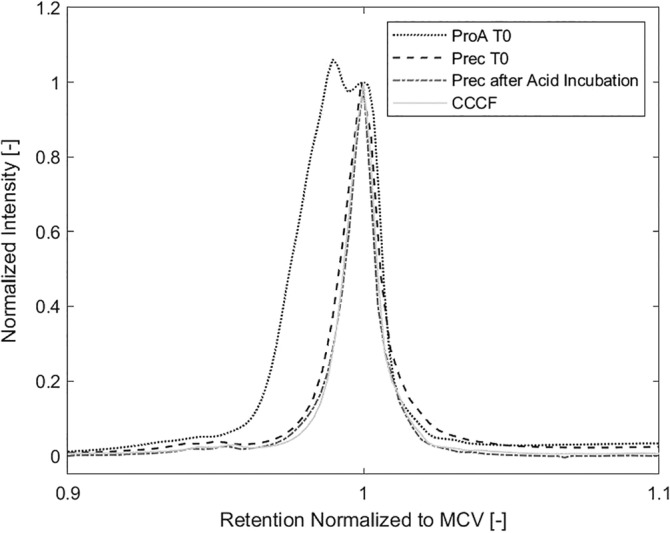
Isoform pattern for protein A purified, precipitated material, precipitated material after acid incubation and CCCF. During protein A chromatography, a transient, acidic variant is formed. CCCF, clarified cell culture fluid

At 5°C (Figure 8), we observe the same trend as at −20°C, the acidic variant in the protein A purified sample turns into the MCV while the precipitated antibody shows a stable isoform pattern over the whole time. For the protein A purified material stored at room temperature, we see the same course that was already described for the storage at −20 and 5°C (Figure 5a), meaning a generation of an additional isoform during protein A chromatography that is not visible anymore after 1 month in storage. After the initial conversion of the acidic isoform to the main charge variant, the pattern stays stable.

For the precipitated material stored at room temperature, the isoform pattern is only stable for 1 month (Figure 5b). After storage of 3 months, we see the appearance of new, acidic isoform and after 6 months, the sample seems to be completely degenerated with new acidic and basic variants present.

#### 3.2.2 Formation of acidic isoform during protein a chromatography

In recent years, multiple groups, including our own, have observed conformational plasticity of antibodies during protein A chromatography.^21^, ^22^, ^23^ In this work we could again observe the formation of a transient antibody isoform. We incubated the T_0_ material after protein A chromatography at three different temperatures (5°C, room temperature and 40°C) for a total of 120 hr and tested the charged variant composition at *T*_0_, 24, 48, and 120 hr.

**Figure 9:**
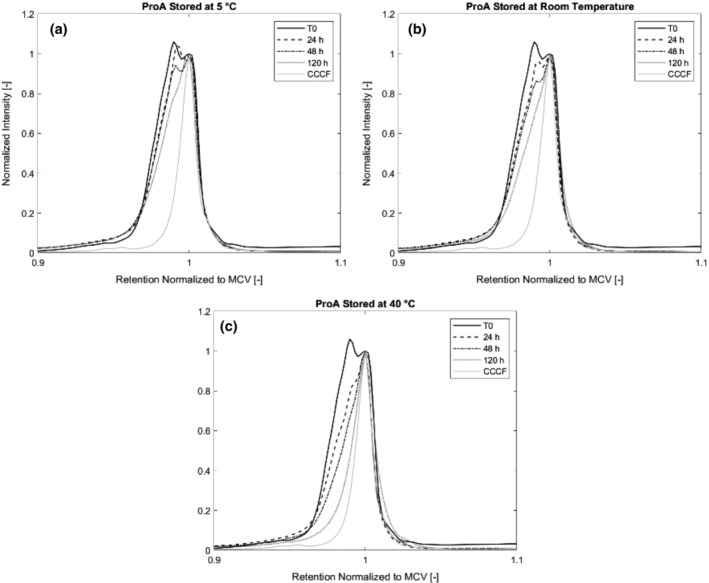
(a–c) Isoform pattern of protein A purified material stored at 5°C, room temperature and 40°C for 120 hr compared to CCCF. Incubation of the protein A purified material with the acidic isoform shows temperature dependent reconversion of the acidic isoform the MCV within a matter of days. CCCF, clarified cell culture fluid

We were able to show that re‐conversion of the acidic isoform to the MCV is temperature dependent. Figure 9a–c shows chromatograms of the pH gradient HPLC of the T_0_ material after protein A purification.

When incubated at 5°C or room temperature, the acidic isoform remains visible for the first 48 hr. Between 48 and 120 hr, it is almost entirely converted to the MCV and just a small shoulder remains visible. Since the yields are stable (see chapter 3.1), we know that the acidic isoforms reforms into the MCV and is not just degraded. At an incubation temperature of 40°C, the acidic isoform is almost entirely gone after 48 hr and not detectable anymore after 120 hr showing that the kinetics are temperature dependent.

To see whether the formation of the acidic isoform results from the low pH that the antibodies are exposed to during elution in protein A chromatography or the subsequent incubation at low pH for viral inactivation. Therefore, we incubated the precipitated antibody after resolubilization in pH 3.6 for 1 hr and performed pH gradient analysis again. As seen in Figure 6, the low pH incubation does not lead to change in isoform pattern, meaning that the structure change seen for protein A purified samples is due to the chromatography itself and not the shift to low pH.

### 3.3 Structural alterations during long‐term storage

**Figure 10:**
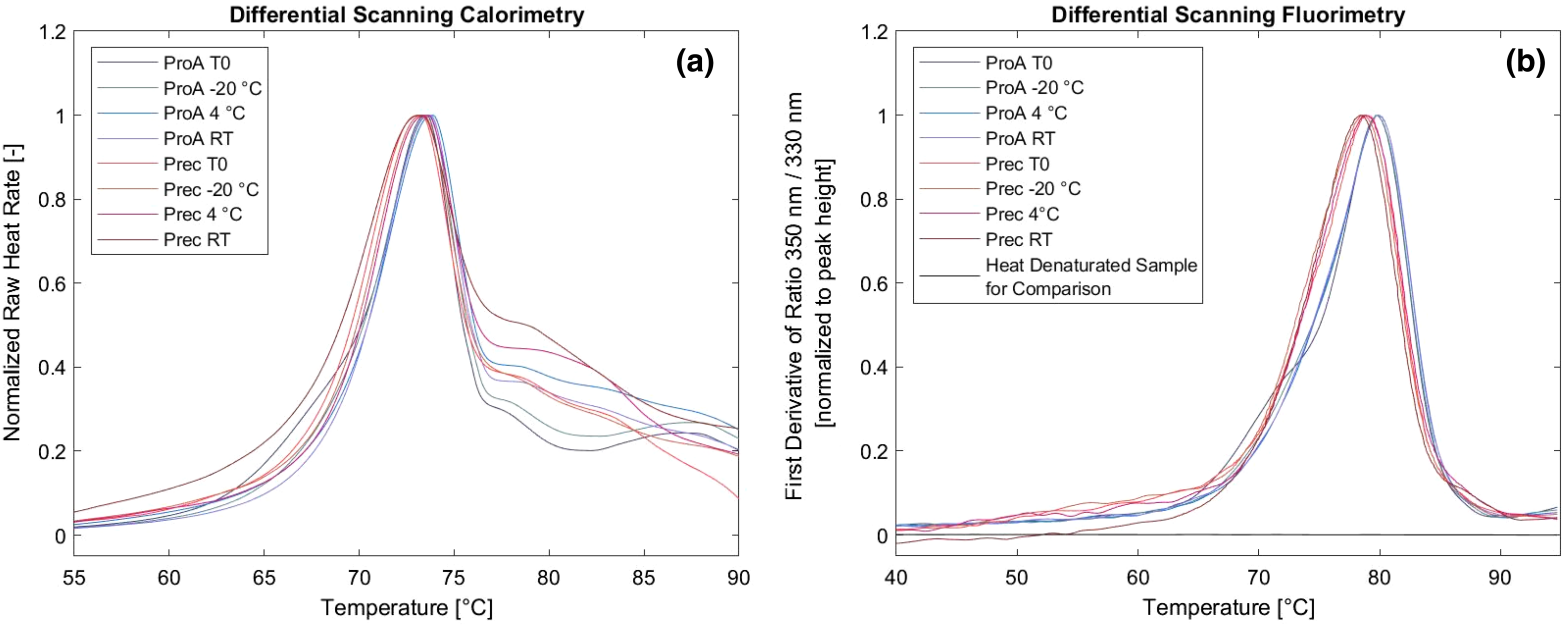
(a) Differential scanning calorimetry and (b) differential scanning fluorimetry spectra for protein A purified and precipitated material at start and end points of storage. Both techniques, DSC and DSF, indicate a slight decrease in stability of the precipitated material

In addition to monomer content, product loss, HMWI, and isoform composition, we wanted to check for possible changes in the structure of the stored antibodies. For this we used DSC, DSF as well as CD. In DSC, unfolding of antibodies follows a biphasic transition where the unfolding of the Fab and CH2 domain occur approximately at the same temperature and overlap in the thermogram. The CH3 domain endures higher temperatures and unfolds a bit later.^24^ On average, the main unfolding event (Fab and CH2) of the protein A purified material lies at 73.7°C while the precipitated material unfolds at 73.3°C (Figure 10a, Table S2).

This indicates that the precipitated material is slightly less stable than the protein A purified one. At higher temperatures, we observe slight differences in the unfolding of the CH3 domains of the antibodies. We noticed that the presence of residual polyethylene glycol influences the DSC signal strongly but this was taken into account by respective blank subtractions. Also the co‐presence of different host cell proteins could impact the thermal stability of the samples. In the differential scanning fluorimetry the inflection temperature of the protein A purified samples is on average at 79.8°C and is therefore about 1°C higher than the one of the precipitated antibodies which is at 78.7°C (Figure 10b, Table S2). Due to the fast heat ramping in the DSF (30°C/min), the unfolding of the antibodies occurs in a single transition rather than a biphasic one as is usually observed in DSC experiments. In both cases (DSC and DSF), the protein A purified sample shows a slightly higher unfolding temperature than the precipitated material. This can be either due to impurities in the precipitation sample adding to the signal, or due to differences in the stability of the antibody itself. To get more insight into the structure and possible structure differences, we measured the CD spectra of precipitated and resolubilized antibody in comparison to protein A purified samples.

**Figure 11:**
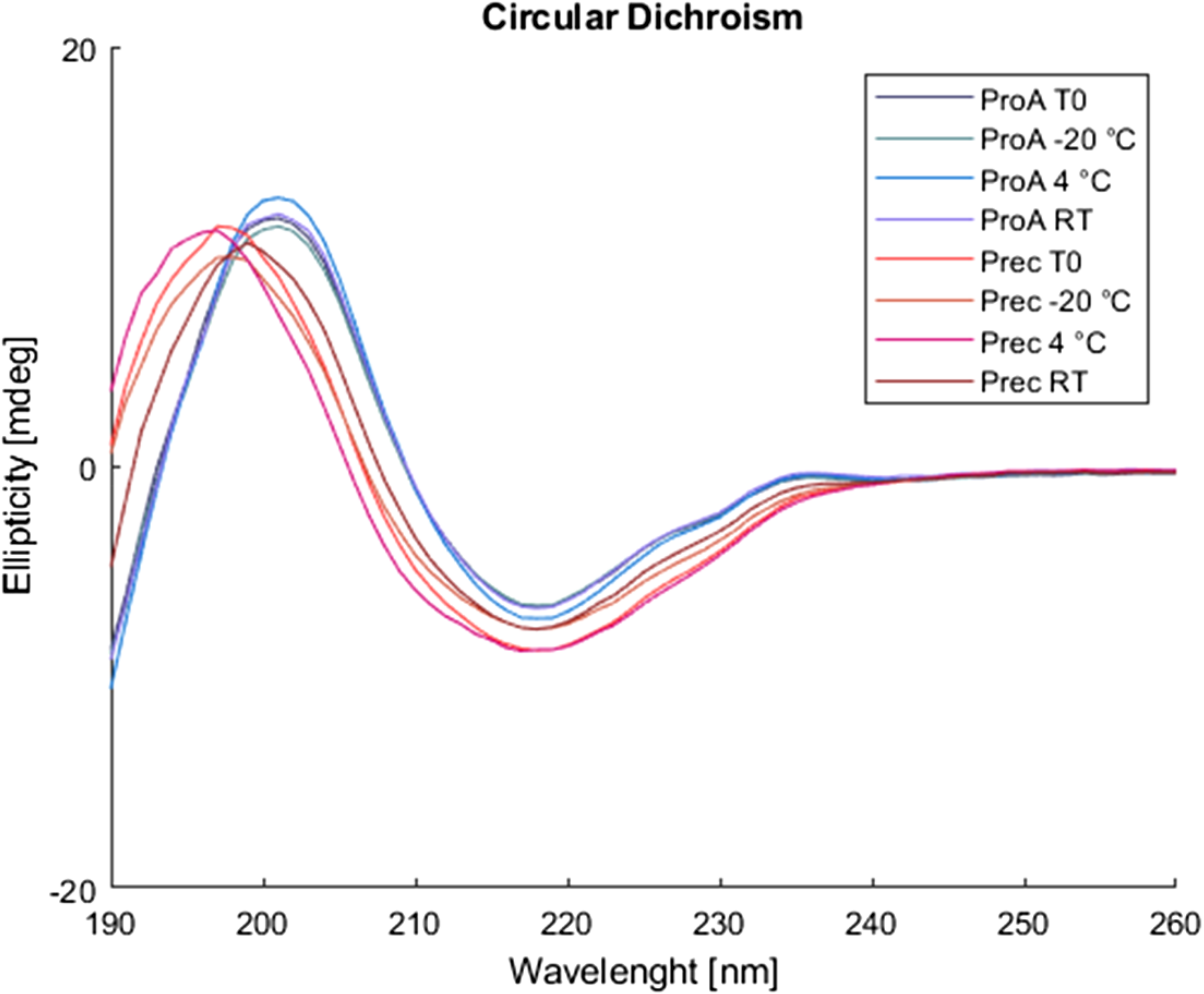
Circular dichroism spectra for protein A purified and precipitated material at start and end points of storage. Deconvolution of the spectra shows that a higher amount of random coil is present

In CD measurements, the precipitated antibodies have an observed maximum at 198 nm while the protein A purified material has its maximum 202 nm (Figure 11). All samples have a minimum at 218 nm with the precipitated antibodies giving a stronger signal than the protein A purified material.

Deconvolution of the spectra shows that the precipitated antibody contains a higher amount of random coil. This could be the reason for the reduced stability observed in DSC and DSF experiments. We performed a series of experiments to rule out the possibility that the changes between the protein A purified and the precipitated material are caused by residual PEG presence or the co‐presence of host cell proteins. Spiking the HCP pool of the precipitated material with protein A purified antibody did not cause a shift in the CD signal as it was observed for the precipitated material. Neither did the spiking of the protein A purified material with PEG concentration between 0.5 to 5%. The resulting data is presented in Figure S1.

## 4 DISCUSSION

In our study we compared protein A purified antibody to precipitated antibody in terms of intermediate stability at different temperatures. For the protein A purified material, the storage temperature had no significant impact on monomer content, HMWI or product loss compared to the starting material. HMWI increased only by 2% during the 13 month storage period which could be easily removed in a polishing step later in the downstream process. The protein A purified material can therefore be stored at −20°C, 5°C, and even room temperature with similar stability.

Precipitated material can be stored at −20 and 5°C but product losses have to be expected if the material is stored for more than 1 month. To obtain yields >90%, it is necessary to reconstitute the antibody within the first month of storage, making precipitate storage an option for intermediate storage before the next process step, but not feasible for long term storage. For the protein A purified material stored at −20°C, 5°C, and room temperature as well as the precipitated material stored at −20 and 5°C, the product losses are counteracted by increases in HMWI. For the precipitated material stored at room temperature, the HMWI start rising only after 6 months but product losses are observed gradually during the whole storage period. Therefore, the initial product losses have to be a result of degradation processes which lead to the formation of LMWI. The decrease in resolubilization yields observed for the precipitated material stored at −20 and 5°C could be due to the formation of compact precipitates which are harder to solubilize. Performing a buffer resolubilization screening for the precipitates stored for longer periods (>3 months) could improve resolubilization yields. After this initial drop, the resolubilization yields remain stable for the residual storage period. Storage at room temperature is not recommended at all as it leads to formation of new isoforms and degradation starts visibly after 1 month.

DSF, DSC, and CD spectra show differences between the protein A purified material and the PEG precipitates. All three methods indicate that the stability of the precipitated material is slightly decreased compared to the protein A purified material. This is in contrast to observations that our group has made earlier with antibodies of the subclass IgG1 that showed no differences based on the capture conditions. Our study shows that antibodies of the IgG2 have a higher susceptibility of structural changes during manufacturing. This could be due to the fact that IgG2 is less flexible than IgG1 as it has a shorter hinge region and contains more inter‐heavy chain disulfide bonds which make it more rigid^25^ and potentially leads to difficulties in the resolubilization process. An additional complication for IgG2 could be more possibilities for disulfide bridge shuffling. An interesting fact is that the degradation was only visible in isoform analysis while the SEC chromatograms lead to believe structural integrity.

It would be very interesting to determine the exact structural nature of precipitates by for example, high‐resolution microscopy in the future to explain these differences in IgG1 and IgG2 behavior. Product alterations could also be followed in unit operations further downstream. Thereby it becomes possible to see if the alterations detected after the capture step have an impact on the final drug product. Due to our analysis, we recommend a broad pallet of analytical tools to track product variations and product alterations. While one method, for instance size exclusion, could lead to the assumption of a stable protein, another method, for instance CD, could show significant changes in the structure of the product. We at least recommend three complementary methods, while one tracks product degradations in size (SEC), one tracks product alterations in structure (CD) and one tracks product alterations in charge (ion exchange). In our case, following the product through the complete process was not possible since the process was not yet fully developed when we evaluated the stability of the capture intermediates. For production, the product alterations have to be tracked through the complete process chain.

Changes in the charged isoform pattern were detected by HPLC‐CIEX with a pH gradient. We observed the formation of an acidic variant during protein A chromatography that slowly converts back into the main charged variant in the first days of storage. The kinetics of these conversions depend on the storage temperature, with a faster conversion for higher temperatures where for storage at 5°C the conversion takes a week, while at elevated temperatures of 40°C it takes only 48 hr. We were also able to show that the formation of this acidic isoform is connected to the chromatography itself. Incubation of the precipitated material at a pH of 3.6 for 1 hr does not lead to a change in the isoform pattern and a formation of this isoform due to the low pH at elution can therefore be ruled out. This confirms the findings of our previous work^21^ where it was shown that the conformational changes occur while the antibodies are adsorbed to the material. Several protein modifications are known to shift the isoelectric point of a protein to the acidic side, most commonly deamidation reactions but also C‐terminal lysine cleavages, sialylation, and oxidation/reduction,^6^ but none of them are known to be completely reversible in the timeframes observed in our experiments. Others have shown that protein A chromatography can lead to significant structural changes in the antibody resulting in reduced hydrodynamic radii.^23^ Such structural changes could be the cause of the phenomenon we see in our experiments, but we observed no change of hydrodynamic radius of the antibody in our samples (data not shown).

In summary, the antibodies had better storage abilities when they were captured by protein A chromatography than by precipitation. The protein A purified material was found to be well storable at all tested temperatures. With the analytics used in this study we could not detect any structural changes within the protein A purified samples over time. Monomer content and product loss were constant over a period of 13 months. The precipitated antibodies stored at room temperature started to degrade after 3 months resulting in an increase of LMWI impurities first which are later followed by the formation of HMWI too. The reduced storability of the precipitated proteins could be due to the higher presence of HCP in the precipitated samples which could include proteases leading to degradation of the product. Especially at room temperature, the precipitated material is prone to aggregate formation as well. At −20 and 5°C, the precipitated material can be stored without any product losses but reconstitution yields suffer if the material is stored for more than 1 month. The working window is however large enough to ship the material to a different production site and conduct experiments on the material mid‐manufacturing or continue processing.

## Supporting information


**Figure S1** Circular dichroism spectra for protein A purified and precipitated material at start. HCPs of a precipitated material were purified and protein A purified sample spiked with the precipitated HCPs. Protein A purified material was also spiked with different PEG concentrations. Addition of HCPs or PEG does not lead to a shift of the CD signal. Please note that the addition of PEG leads to a saturation of the signal <200 nm.
**Table S1** Monomer content (%), HMWI (%) and product loss (%) for storage at ‐20 °C, 5 °C and room temperature.
**Table S2** Unfolding temperatures of main unfolding event in DSC and the inflection temperature in DSF experiments in the protein A purified and precipitated material.
