# Supplementary material for: Mid‐manufacturing storage: Antibody stability after chromatography and precipitation based capture steps
Source: Biotechnol Prog. 2019 Nov 1;36(2):e2928. doi: 10.1002/btpr.2928 (PMC7187330; doi:10.1002/btpr.2928)
Supplement: Supplementary file 1 — Figure S1 Circular dichroism spectra for protein A purified and precipitated material at start. HCPs of a precipitated material were purified and protein A purified sample spiked with the precipitated HCPs. Protein A purified material was also spiked with different PEG concentrations. Addition of HCPs or PEG does not lead to a shift of the CD signal. Please note that the addition of PEG leads to a saturation of the signal <200 nm. Table S1 Monomer content (%), HMWI (%) and product loss (%) for storage at ‐20 °C, 5 °C and room temperature. Table S2 Unfolding temperatures of main unfolding event in DSC and the inflection temperature in DSF experiments in the protein A purified and precipitated material. [file BTPR-36-e2928-s001.docx]

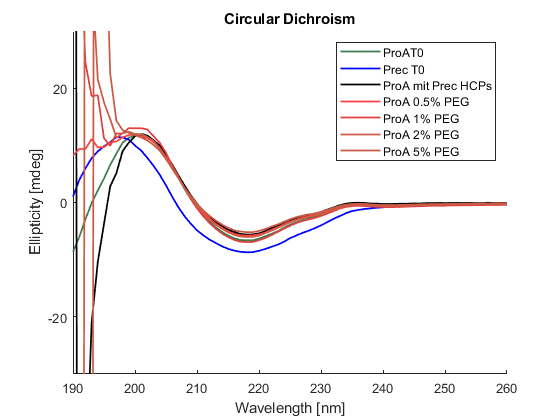


Supplementary Figure 1: Circular dichroism spectra for protein A purified and precipitated material at start. HCPs of a precipitated material were purified and protein A purified sample spiked with the precipitated HCPs. Protein A purified material was also spiked with different PEG concentrations. Addition of HCPs or PEG does not lead to a shift of the CD signal. Please note that the addition of PEG leads to a saturation of the signal <200 nm.

*Supplementary Table 1: Monomer content (%), HMWI (%) and product loss (%) for storage at -20 °C, 5 °C and room temperature.*

| Storage at -20 °C | | | | | | |
| --- | --- | --- | --- | --- | --- | --- |
|  | Monomer Content ± St.Dev. (%) | | HMWI ± St.Dev. (%) | | Concentration compared to T_0_ ± St.Dev. (%) | |
|  | ProA Purified Material | Precipitated Material | ProA Purified Material | Precipitated Material | ProA Purified Material | Precipitated Material |
| At T_0_ | 99.8 ± 0.2 | 85.2 ± 3.6 | 0.58 ± 0.08 | 5.54 ± 0.68 | 100.0 ± 0.69 | 100.0 ± 0.88 |
| At T1M | 99.5 ± 0.2 | 85.5 ± 3.6 | 0.75 ± 0.02 | 5.93 ± 0.66 | 100.5 ± 1.13 | 90.2 ± 1.42 |
| At T3M | 97.8 ± 1.4 | 83.1 ± 0.4 | 1.41 ± 0.20 | 5.74 ± 0.16 | 99.3 ± 0.30 | 77.7 ± 7.16 |
| At T6M | 98.0 ± 0.4 | 83.2 ± 1.6 | 1.76 ± 0.04 | 5.12 ± 0.19 | 96.8 ± 0.68 | 73.0 ± 1.95 |
| At T10M | 99.1 ± 0.2 | 82.9 ± 0.4 | 2.54 ± 0.28 | 6.42 ± 0.10 | 100.7 ± 0.23 | 72.6 ± 2.66 |
| At T13M | 98.2 ± 0.3 | 84.3 ± 1.8 | 2.20 ± 0.28 | 5.6 ± 0.04 | 100.0 ± 0.23 | 67.3 ± 3.45 |
|  | | | | | | |
| Storage at 5 °C | | | | | | |
|  | Monomer Content ± St.Dev. (%) | | HMWI ± St.Dev. (%) | | Concentration compared to T_0_ ± St.Dev. (%) | |
|  | ProA Purified Material | Precipitated Material | ProA Purified Material | Precipitated Material | ProA Purified Material | Precipitated Material |
| At T_0_ | 99.8 ± 0.2 | 85.2 ± 3.6 | 0.58 ± 0.08 | 5.54 ± 0.68 | 100.0 ± 0.69 | 100.0 ± 0.88 |
| At T1M | 100.0 ± 0.0 | 84.8 ± 1.8 | 0.81 ± 0.06 | 6.05 ± 0.24 | 97.9 ± 0.67 | 97.0 ± 3.98 |
| At T3M | 97.5 ± 0.4 | 80.4 ± 2.4 | 2.21 ± 0.44 | 5.89 ± 0.58 | 100.4 ± 3.42 | 68.4 ± 4.59 |
| At T6M | 97.9 ± 0.0 | 82.0 ± 1.6 | 2.00 ± 0.40 | 4.89 ± 0.20 | 98.6 ± 0.04 | 70.5 ± 2.13 |
| At T10M | 98.2 ± 0.8 | 77.7 ± 0.4 | 2.32 ± 0.64 | 5.87 ± 0.48 | 102.4 ± 1.82 | 71.4 ± 0.25 |
| At T13M | 98.3 ± 0.6 | 79.8 ± 1.6 | 2.32 ± 0.26 | 6.24 ± 0.02 | 99.2 ± 0.64 | 66.5 ± 1.91 |
|  | | | | | | |
| Storage at Room Temperature | | | | | | |
|  | Monomer Content ± St.Dev. (%) | | HMWI ± St.Dev. (%) | | Concentration compared to T_0_ ± St.Dev. (%) | |
|  | ProA Purified Material | Precipitated Material | ProA Purified Material | Precipitated Material | ProA Purified Material | Precipitated Material |
| At T_0_ | 99.8 ± 0.2 | 85.2 ± 3.6 | 0.58 ± 0.08 | 5.54 ± 0.68 | 100.0 ± 0.69 | 100.0 ± 0.88 |
| At T1M | 99.4 ± 0.8 | 79.0 ± 3.6 | 1.05 ± 0.04 | 5.06 ± 0.32 | 98.9 ± 1.16 | 94.8 ± 3.95 |
| At T3M | 97.3 ± 0.0 | 74.0 ± 5.6 | 2.03 ± 0.18 | 4.40 ± 0.62 | 99.0 ± 1.17 | 85.9 ± 6.23 |
| At T6M | 96.7 ± 0.4 | 70.1 ± 7.2 | 2.75 ± 0.02 | 4.33 ± 0.52 | 100.6 ± 1.40 | 95.4 ± 1.29 |
| At T10M | 97.7 ± 0.2 | 64.0 ± 4.2 | 2.54 ± 0.28 | 7.44 ± 5.14 | 99.8 ± 0.97 | 104.2 ± 0.49 |
| At T13M | 98.0 ± 0.4 | 60.5 ± 2.2 | 2.54 ± 0.18 | 15.57 ± 0.20 | 101.4 ± 0.30 | 119.0 ± 1.33 |

Supplementary Table 2: Unfolding temperatures of main unfolding event in DSC and the inflection temperature in DSF experiments in the protein A purified and precipitated material.

| **Sample** | **Unfolding Temperature [°C] in DSC (Fab and CH2 domain)** | **Inflection Temperature [°C] in DSF** |
| --- | --- | --- |
| **ProA T_0_** | 73.5 | 79.8 |
| **ProA -20 °C** | 73.7 | 79.8 |
| **ProA 5 °C** | 73.9 | 79.8 |
| **ProA RT** | 73.7 | 79.8 |
| **Prec T_0_** | 73.1 | 78.9 |
| **Prec -20 °C** | 73.4 | 78.7 |
| **Prec 5 °C** | 73.6 | 78.9 |
| **Prec RT** | 73.3 | 78.4 |
